# Genome-Wide Analysis of the NAC Family Associated with Two Paleohexaploidization Events in the Tomato

**DOI:** 10.3390/life12081236

**Published:** 2022-08-15

**Authors:** Jiale Yuan, Ying Liu, Zhenyi Wang, Tianyu Lei, Yanfang Hu, Lan Zhang, Min Yuan, Jinpeng Wang, Yuxian Li

**Affiliations:** 1 Center for Genomics and Computational Biology, School of Life Sciences, North China University of Science and Technology, Tangshan 063000, China; 2 University of Chinese Academy of Sciences, Beijing 100049, China

**Keywords:** paleohexaploidization, tomato, NAC transcription factor, fruit ripening

## Abstract

NAC transcription factors play an important regulatory role in tomato fruit ripening. We chose a novel perspective to explore the traces left by two paleopolyploidizations in the NAC family using a bioinformatics approach. We found that 85 (*S. lycopersicum*) and 88 (*S. pennellii*) members of the NAC family were present in two tomatoes, and most of them were amplified from two paleohexaploidizations. We differentiated NAC family members from the different paleohexaploidizations and found that the SWGT-derived *NAC* genes had more rearrangement events, so it was different from the DWGT-derived *NAC* genes in terms of physicochemical properties, phylogeny, and gene location. The results of selection pressure show that DWGT-derived *NAC* genes tended to be positively selected in *S. lycopersicum* and negatively selected in *S. pennellii*. A comprehensive analysis of paleopolyploidization and expression reveals that DWGT-derived *NAC* genes tend to promote fruit ripening, and are expressed at the early and middle stages, whereas SWGT-derived *NAC* genes tend to terminate fruit growth and are expressed at the late stages of fruit ripening. This study obtained *NAC* genes from different sources that can be used as materials for tomato fruit development, and the method in the study can be extended to the study of other plants.

## 1. Introduction

The tomato (*Solanum lycopersicum*, abbreviated *S. lycopersicum*) is a diploid (2n = 24) crop of the Solanaceae family whose fruits are rich in organic nutrients such as sugars, amino acids, lycopene, β-carotene, and vitamin C (ascorbic acid), and is one of the important economic vegetable crops in the world [1,2]. The tomato is a model organism for agronomic, nutritional, and genetic studies due to its small genome (~900 Mb) [3], short growth cycle, high self-fertility, and homozygosity [4,5,6].

Various factors such as plant hormones, environmental signals, and transcription factors influence fruit ripening in tomato, of which the NAC transcription factor is one of the major ones [7,8]. The NAC family, named after *NAM*, *ATAF1/2*, and *CUC2*, is one of the largest families of TFs that play important roles in different stages of plant growth, development, and maturation [8,9]. An increasing number of studies revealed that NAC proteins play important roles in various metabolic pathways, and resistance to biotic and abiotic stresses in tomatoes, such as flower border formation [10], leaf senescence [11], fruit ripening [12,13,14,15], phytohormones [16], salt [17], drought [17,18], and heavy metals [19].

Paleopolyploidization, also known as whole-genome duplication (WGD), is an important driver of plant evolution through the numerical multiplication and functional diversification of genes [20,21,22,23,24]. The tomato genome has undergone two paleohexaploidizations in its evolutionary history, also known as whole genome triploidization (WGT) events. The first was a γ event [3] (~120 mya) that occurred in the common ancestor of dicotyledons, referred to here as DWGT. The second was a paleohexaploidization (~67 mya) that occurred in the common ancestor of Solanaceae [3,25,26], referred to here as SWGT. Paleopolyploidization produces more gene family members that may affect the regulatory functions of the gene family, and influence the growth and development of the crop. For example, the effects of paleopolyploidization on gene function were identified in the studies of several gene families such as *PHYB1/PHYB2* [3], *U-box E3* gene [27], XTH and DOF [28], but few studies have associated tomato paleohexaploidization with its NAC family characteristics.

Given the importance of the NAC family in the physiological activity of tomatoes, this study presents a novel comparative analysis of the NAC family and paleohexaploidizations in two tomato genomes (*S. lycopersicum* and its wild-type *S. pennellii*). This study uses *Coffea canephora* [29] as an out-group that did not undergo SWGT to distinguish *NAC* genes generated by different paleohexaploidizations. Furthermore, the study explores the associative features of two paleohexaploidizations with the NAC family in terms of chromosomal localization, physicochemical properties, phylogeny, selection pressure, differential expression, and functional enrichment. This study helps in applying the characteristics of the NAC family to the improvement of tomato fruits.

## 2. Materials and Methods

### 2.1. Species Selection and the Generation of NAC Members

Two tomato genomic data were downloaded from Sol Genomics Network (https://solgenomics.net/, accessed on 8 July 2022) [30], with ITAG version 4.0 for cultivar *S. lycopersicum* [31] and Spenn-v2.0 for the wild species *S. pennellii* [32]. Genomic data for out-group *C. canephora* [29] were downloaded from the Coffee Genome Hub (https://coffee-genome-hub.southgreen.fr/, accessed on 8 July 2022). Hidden Markov model (HMM) seeds for the NAC domain (PF02365) were downloaded from Pfam [33]. Bioperl [34] was used to manipulate sequences, Muscle [35] to perform multiple sequence alignment, and HMMER 3 [36] to perform Pfam Hmm seed-based sequence alignment. The threshold for specific NAC library construction was 1 × 10${}^{-20}$, and the threshold for *NAC* identification was 1 × 10${}^{-10}$.

### 2.2. Physical Localization, Physicochemical Properties, and Phylogenetic Analysis

The distribution of *NAC* members on chromosomes was demonstrated using MG2C [37] on the basis of the location of genes in genomic assembly data. The primary structure of NAC proteins was analyzed using Protparam in Expasy [38]. The subcellular localization of NAC proteins was analyzed using CELLO [39]. The secondary structure of NAC proteins was predicted using SOPMA [40]. The multiple sequence alignment of the identified NAC families was performed using Muscle software, and the construction of evolutionary trees using the maximal likelihood method (ML) in FastTree [41] software setting Bootstrap = 1000. The evolutionary tree was trimmed and embellished using the Chiplot tool (https://www.chiplot.online/, accessed on 8 July 2022).

### 2.3. Genomic Collinearity Search and Selection Pressure Analysis

The search for collinear fragments was performed in *C. canephora* and two tomato genomes using MCScanX [42] with default parameters to obtain genes of different origins, such as tandem repeats and paleopolyploidization. The origin of the *NAC* genes was obtained by querying the identified NAC family members in the above results. On the basis of the above results, the downstream analysis of MCScanX (detect_collinearity_within_gene_families.pl) was executed to obtain the collinearity of NAC family members. The multiple sequence alignment of collinear *NAC* genes using Clustalw [43] software, followed by calculation of Ka (nonsynonymous substitutions) and Ks (synonymous substitutions) values for each collinear *NAC* gene pair using Bioperl. The selection pressure on *NAC* genes of different species was analyzed on the basis of the ratio of Ka to Ks, with Ka/Ks > 1 being positive selection, Ka/Ks = 1 being neutral selection, and Ka/Ks < 1 being purifying selection. Genomic collinearity and *NAC* genes were visualized using Circos [44].

### 2.4. Fruit-Ripening-Specific Expression Analysis and Enrichment Analysis

For the analysis of differential expression and enrichment in the NAC family, we selected the expression microarray data of tomato (cv., Money Maker) at three fruit-ripening stages. The three stages were mature green (MG), breaker (Br), and breaker + 10 days (Br10), and each stage had three biological replicates. These data were downloaded from the NCBI Gene Expression Omnibus [45] and stored in accession number GSE108415 [46]. Limma [47] software screens for specific differentially expressed genes in fruit ripening. AnnotationHub [48], DOSE [49], and clusterProfiler [50] were used for the enrichment analysis of differentially expressed genes and the NAC family. ggplot2 [51] and enrichplot [52] were used to visualize differential expression volcano maps and enrichment analysis results. pheatmap (https://github.com/raivokolde/pheatmap, accessed on 8 July 2022) was used for clustering and drawing heat maps. Linear fit models for differentially expressed genes were estimated using the least-squares method. The *p*-value and adj. *p*-Value for each group comparison were calculated using the modified t-statistic and BH method [53], respectively. The screening threshold for differential genes was |logFoldChange| > 2 and *p*-value < 0.05. The screening threshold for both GO and KEGG enrichment was adj. *p*-value < 0.05. The FoldEnrichment value in Go enrichment analysis is equal to the ratio of the GeneRatio to BgRatio; RichFactor in KEGG enrichment analysis is equal to the ratio of molecules of GeneRatio (i.e., Count) to molecules of BgRatio.

## 3. Results

### 3.1. Identification and Localization of NAC Family

*C. canephora* has undergone only one paleohexaploidization (DWGT) event and has a relatively conservative evolutionary rate, thus serving as an out-group species. The *NAC* genes in *C. canephora*, *S. lycopersicum*, and *S. pennellii* were identified on the basis of a hidden Markov model of the NAC domain. The number of *NAC* genes in each species was recorded (Table 1). The results show that *S. lycopersicum* and *S. pennellii* had similar numbers of *NAC* genes (85 vs. 88). The number of *NAC* genes was significantly higher in both tomatoes when compared to *C. canephora*. This suggests that the second paleohexaploidization may have resulted in the expansion of the NAC family members in the tomato genome.

**Figure 1:**
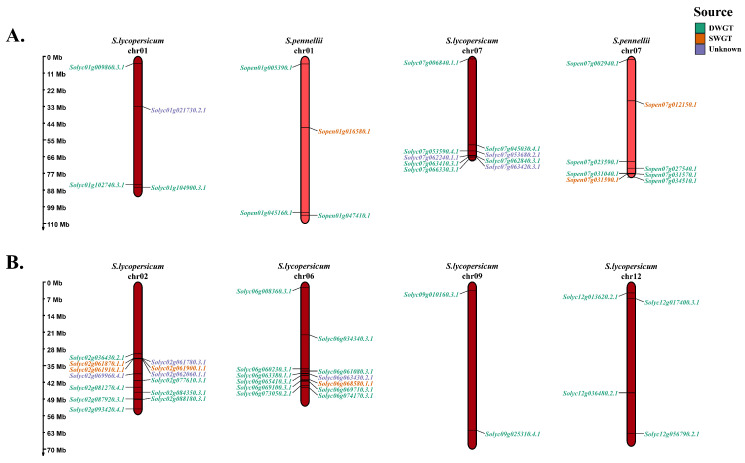
Distribution of *NAC* genes on two tomato chromosomes. (**A**) *NAC* on chromosomes 1 and 7 of *S. lycopersicum* and *S. pennellii* with a similar positional distribution. (**B**) *NAC* on different chromosomes of *S. lycopersicum* with chromosome end distribution. Each *NAC* gene is labeled with its possible origin: DWGT, SWGT, and unknown.

The physical localization of *NAC* genes in *S. lycopersicum* and *S. pennellii* shows that *NAC* genes were distributed very similarly on the same chromosomes in different tomatoes (Figure 1A). For example, *NAC* genes had the same arrangement on chromosome 1 in both tomatoes. On chromosome 7, the difference was that *S. pennellii* had one more gene located in the middle of the chromosome, *Sopen07g012150.1*. The results for different chromosomes show that *NAC* genes are unevenly arranged on different chromosomes (Figure 1B). For example, the number of *NAC* genes on chromosomes 2 and 6 was more than that on chromosomes 9 and 12. At the overall level, *NAC* genes often form some gene clusters at the ends of the chromosomes (Figure S1). For example, the average distance of *NAC* genes on chromosome 2 of *S. lycopersicum* was 0.0578 Mb (*Solyc02g061780.3.1*, *Solyc02g061900.1.1*, *Solyc02g062060.1.1*, *Solyc02g061870.1.1* and *Solyc02g061910.1.1*), which is very close (Figure S1A). Similarly, *NAC* genes also form clusters on chromosome 4 of *S. pennellii*, with an average distance of 0.0586 Mb (Figure S1B).

### 3.2. Physicochemical Characteristics of NAC Proteins

**Figure 2:**
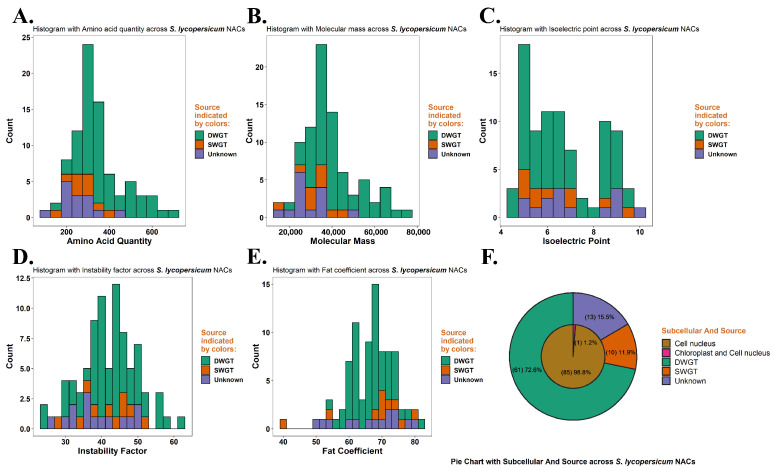
Primary structural and physicochemical properties of NAC proteins. (**A**) The number of amino acids of NAC protein in *S. lycopersicum* ranged from 122 to 687. (**B**) The relative molecular mass of NAC protein in *S. lycopersicum* ranged from 14,427.78 to 76,250.64, a similar to the distribution of amino acid numbers. (**C**) The isoelectric points of NAC proteins in *S. lycopersicum* ranged from 4.58 to 9.90, and they were mostly acidic. (**D**) The instability coefficients of NAC proteins in *S. lycopersicum* ranged from 24.28 to 61.13, and most of them belonged to instability coefficients (>40). (**E**) The fatty coefficients of NAC proteins in *S. lycopersicum* ranged from 40.45 to 82.39. (**F**) The subcellular localization of NAC proteins in *S. lycopersicum* was mainly in the nucleus. Each NAC protein is labeled with its possible origin: DWGT, SWGT, and unknown.

In total, 173 *NAC* genes were identified in two tomatoes (Table 1), and the primary structure and physicochemical properties of their proteins were analyzed using the Protparam tool (Figure 2 and Figure S2, and Table S1). The results of amino acid numbers show that the majority of NAC proteins had amino acid numbers between 100 and 700, with Solyc03g062750.1.1 having the lowest number of amino acids (122) in *S. lycopersicum*. In addition, two NAC proteins in *S. pennellii* had amino acid numbers greater than 700 (Table S1), Sopen10g022760.1 (853), and Sopen02g025910.1 (1014). The molecular mass of NAC proteins showed a positive correlation with the number of amino acids. In detail, the majority of NAC proteins had molecular masses less than 80,000 (Figure 2B). The NAC protein with the smallest molecular mass was Solyc03g062750.1.1 (14,427.78), which also had the lowest number of amino acids. The NAC protein with the largest molecular mass was Sopen02g025910.1 (115,206.37), which also had the highest number of amino acids (Figure S2 and Table S1).

The results of isoelectric points show that the range of isoelectric points of NAC proteins in *S. lycopersicum* was between 4.58 and 9.90. Among these NAC proteins, (PI < 6.5) 47 were acidic, (PI > 7.5) 26 were alkaline, and the remaining 12 were between 6.5 and 7.5 (Figure 2C and Table S1). Correspondingly, the isoelectric points of NAC proteins in *S. pennellii* ranged from 4.21 to 9.82. Among them, the numbers of acidic and basic proteins were 51 and 26, respectively, and the remaining 11 were neutral proteins (Figure S2 and Table S1). The instability index results show that most of the proteins in two tomatoes (111) had instability coefficients greater than 40, i.e., unstable proteins, and a small number of proteins (62) were less than 40, i.e., stable proteins (Figure 2D and Figure S2 and Table S1). The results of the fat coefficients show that most of the NAC proteins had fat coefficients between 40 and 80 in two tomatoes (Figure 2E and Figure S2, and Table S1).

**Figure 3:**
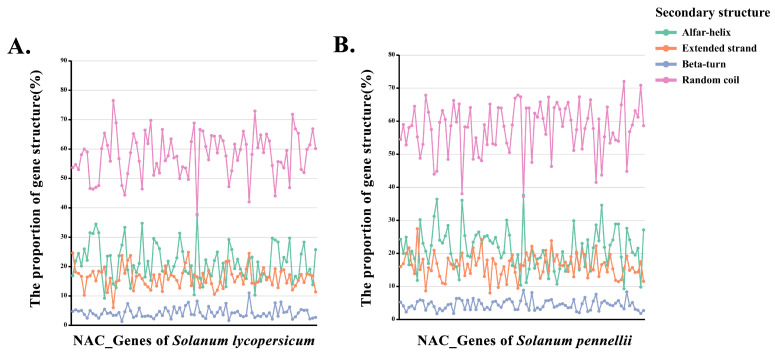
Predicted secondary structures of two tomato NAC proteins. Horizontal coordinates are the sequentially arranged NAC proteins, and vertical coordinates are the proportion of the four secondary structures of each NAC protein. (**A**) Secondary structure of the NAC protein in *S. lycopersicum* has a higher frequency of random curl. (**B**) Secondary structures of NAC proteins in *S. pennellii* are similar in distribution to those of *S. lycopersicum*.

The results of subcellular localization analysis of 173 NAC proteins show that all proteins were present in the nucleus except Solyc04g078670.3.1 and Sopen10g022760.1, which may also be present in chloroplasts (Figure 2F, Table S1). On the basis of the results of secondary structure prediction for 173 NAC proteins, we counted the frequency of secondary structures (e.g., α-helix, β-turn, random coil, and extended chain) for each protein (Figure 3, Table S2). The results show that the random coil had the highest frequency in the NAC proteins and was the dominant structure in the secondary structure. In addition, the frequency of β-turn was the lowest, and the frequency of the α-helix and extended strand was moderate.

### 3.3. Phylogeny of the NAC Family

**Figure 4:**
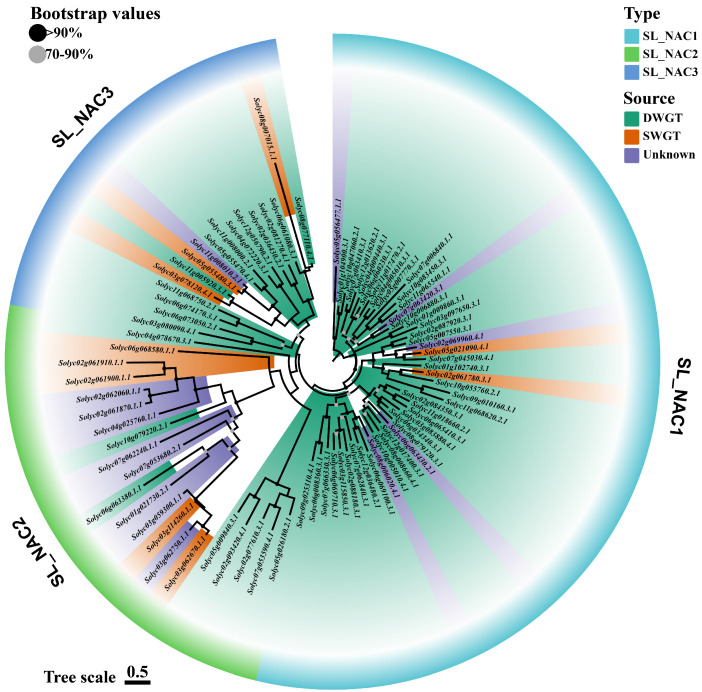
Phylogenetic tree of *S. lycopersicum* NAC proteins, the evolutionary rate of each protein is distributed in blocks. type represents different subgroups; Source represents different sources of *NAC* genes; Bootstrap values are the confidence level of the evolutionary tree; Tree scale represents the unit of branch length.

Phylogenetic trees were constructed in *C. canephora*, *S. lycopersicum*, and *S. pennellii* using Fastree on the basis of the results of the multiple sequence alignment of NAC proteins (Figure 4, Figures S3 and S4). In the case of *S. lycopersicum*, a phylogenetic tree of 85 NAC family members was constructed (Figure 4). According to the topology of the phylogenetic tree, the NAC family can be divided into three subgroups, SL_NAC1 (47 genes), SL_NAC2 (21 genes), and SL_NAC3 (17 genes). Topological results show that SL_NAC2 and SL_NAC1 had the highest and lowest overall evolutionary rates, respectively, with the branching SL_NAC3 having the middle evolutionary rate. This may indicate that more than half of NACs in *S. lycopersicum* are relatively conserved. In addition, Solyc08g007015.1 in SL_NAC3 is noteworthy for its rate of evolution, which was much higher than that of other proteins in the group. Similar to *S. lycopersicum*, the phylogenetic tree of NAC protein can be divided into three subgroups in *S. pennellii*, while the phylogenetic tree of *C. canephora* had no distinct grouping characteristics (Figures S3 and S4).

### 3.4. Association of Two Paleohexaploidizations with NAC Family Amplification

**Figure 5:**
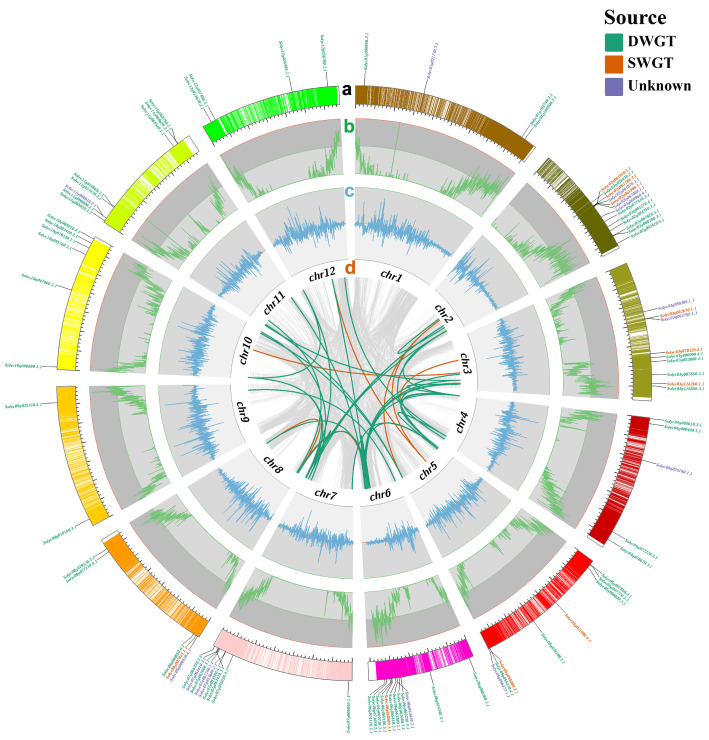
Collinear mapping of *S. lycopersicum NAC* genes. (**a**) *S. lycopersicum* chromosomes and *NAC* genes. (**b**) Distribution of genes on chromosomes (per 100 Kb). (**c**) Distribution of GC content on chromosomes (per 100 Kb). (**d**) Linkages between all collinear gene pairs of *S. lycopersicum* (gray), and collinear linkages of *NAC* genes from different sources (DWGT, red; SWGT, green).

The tomato has undergone two paleohexaploidizations in its evolutionary history, which may have resulted in the expansion of the gene families [54]. The results of whole-genome collinearity analysis show (Table 2) that the genes derived from DWGT accounted for 35.61% of the total genes in *C. canephora*, while those derived from tandem were only 2.58%. This indicates that DWGT is the main driving force of coffee gene amplification. In the genomes of *S. lycopersicum* and *S. pennellii*, genes derived from two paleohexaploidizations contributed 51.79% and 71.31%, which means that DWGT and SWGT are the main amplification methods in the two tomato genomes (Table 2). In contrast, the number of SWGT amplified genes in *S. pennellii* was significantly higher than that in *S. lycopersicum*. In addition, tandem partially contributed to the genomic amplification of two tomato species, adding 11.77% and 15.44% genes. The amplification of the NAC family showed a similar pattern (Table 3). In the case of *S. lycopersicum*, the number of *NAC* genes generated by DWGT, SWGT, and tandem was 61 (71.76%), 10 (11.76%), and 3 (3.53%), respectively. Similarly, paleohexaploidization was the primary mode of NAC family amplification in *C. canephora* and *S. pennellii*, and tandem produced relatively few NAC family members, which is consistent with genomic amplification (Figure 5, Figures S5 and S6).

### 3.5. Selection Pressure on *NAC Genes*

To a certain extent, the ratio of Ka to Ks can reflect the direction of the selection pressure on the genes of the species [55]. According to the collinear relationship between genes within a species, the Ka and Ks between genes in each species were calculated; then, the selection pressure (Ka/Ks) was analyzed and counted (Table 4, Tables S3 and S4). The results show that the number of Ks > 1 (1546) between DWGT gene pairs in *S. lycopersicum* was greater than that of Ks < 1 (778), and the number of Ks > 1 (10) between *NAC* gene pairs produced by DWGT was also greater than that of Ks < 1 (4). This indicates that the positive selection pressure was greater than the negative selection pressure on the genes generated by DWGT, and the *NAC* genes are in the same direction. The number of gene pairs Ks > 1 (363) produced by SWGT was very close to Ks < 1 (339), which indicates that the positive and negative selections of genes produced by SWGT were relatively balanced, and the direction of its selection pressure could not be accurately determined. In the *S. pennellii* genome, genes generated by SWGT were under more pressure from negative selection than from positive selection. Genes generated by DWGT (including *NAC* genes) were under more negative selection pressure than positive selection pressure, which was opposite to *S. lycopersicum*.

### 3.6. Developmental-Stage-Specific Expression Analysis

**Figure 6:**
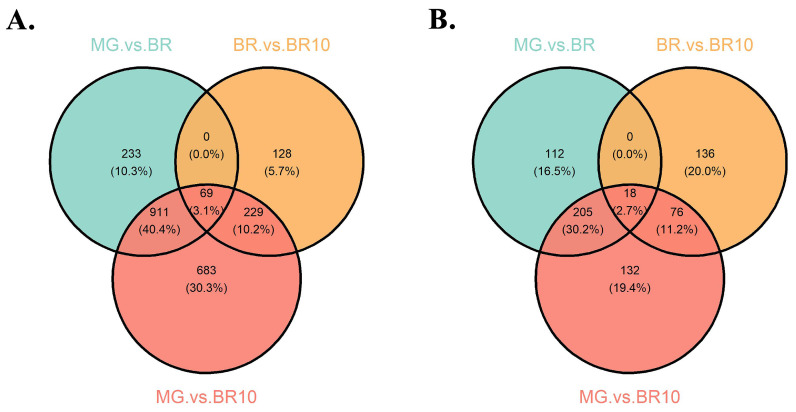
Venn diagram of differentially expressed genes at different maturation stages of *S. lycopersicum*. (**A**) Comparison of the intersection of upregulated genes in the three maturation stages of *S. lycopersicum*. (**B**) Comparison of the intersection of downregulated genes in the three maturation stages of *S. lycopersicum*.

**Figure 7:**
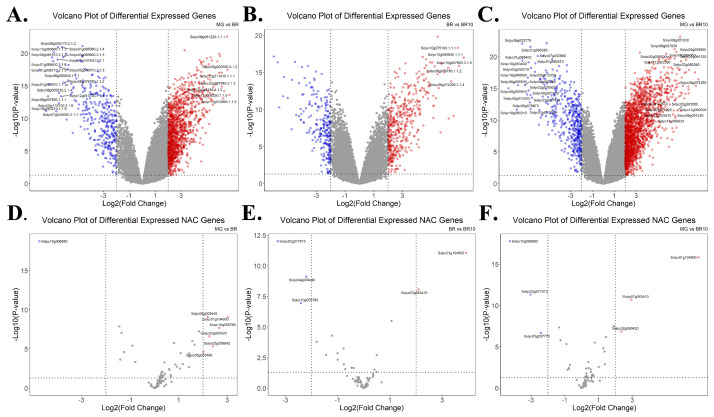
Volcano plot of differential expression at different ripening stages of *S. lycopersicum*. The horizontal coordinate is the log2 value of Fold Change and the vertical coordinate is the −Log10 value of *p*-value, highly significant differentially expressed genes (Log2(Fold Change) absolute value > 6 and −Log10(*p*-value) > 10)) are labeled with gene ID. (**A**) Differential expression of *S. lycopersicum* genes in MG vs. BR phase, up-regulated genes (red), down-regulated genes (blue). (**B**) Differential expression of *S. lycopersicum* gene at BR vs. BR10 stage. (**C**) Differential expression of *S. lycopersicum* gene in MG vs. BR10 phase. (**D**) Differential expression of *S. lycopersicum NAC* gene at MG vs. BR stage. (**E**) Differential expression of *S. lycopersicum NAC* gene at BR vs. BR10 stage. (**F**) Differential expression of *S. lycopersicum NAC* gene at MG vs. BR10 stage.

On the basis of expression data of three maturation stages in *S. lycopersicum*, pairwise comparisons were performed (MG vs. BR, BR vs. BR10, MG vs. BR10), and the statistics of differentially expressed genes were screened (Figure 6, Tables S5 and S6). The results show that the number of upregulated genes shared by the three stages was 69, which was higher than the number of downregulated genes (18), but they had a similar overall proportion (3.1% and 2.7%). Another commonality is that there were no genes in common in either up- or downregulated gene comparisons in the MG vs. BR combination and the BR vs. BR10 combination (Figure 6). The results of differential expression analysis at different maturation stages show (Figure 7) that some differentially expressed genes were highly significant (see Section 2). For example, in the MG and BR combination (Figure 7A), there were 25 highly significantly differentially expressed genes, of which 7 were upregulated and 18 were downregulated. In the BR and BR10 combination, there were only 5 highly significantly differentially expressed genes and all of them were upregulated (Figure 7B). There were 35 highly significantly differentially expressed genes in the MG vs. BR10 combination, of which 16 were upregulated and 19 were downregulated (Figure 7C).

**Figure 8:**
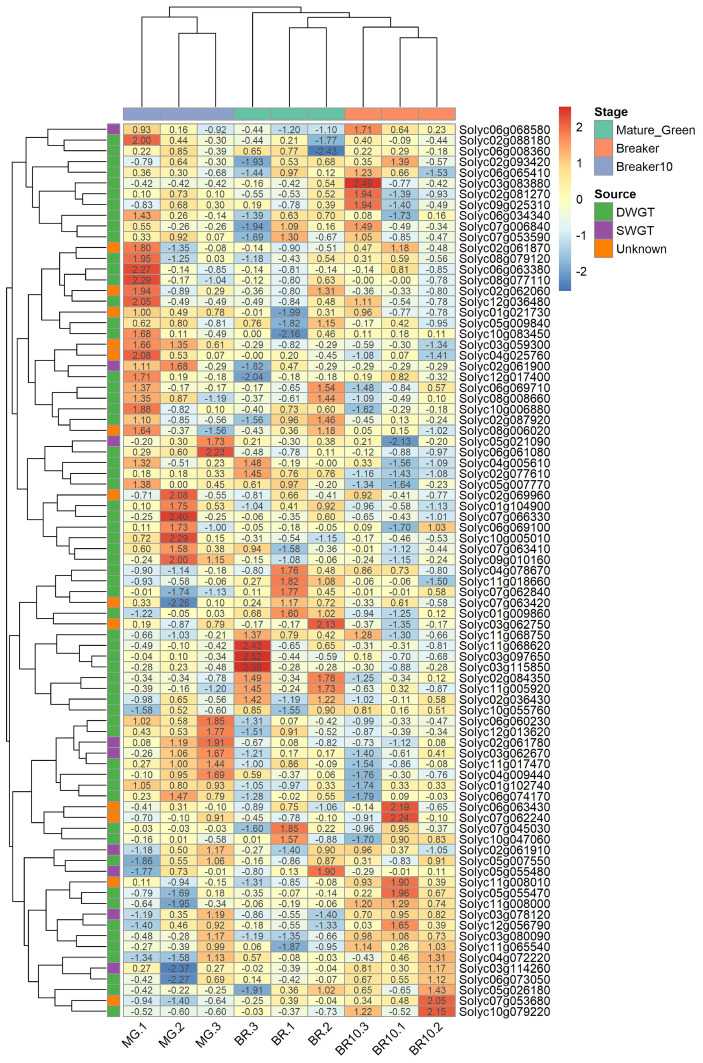
Clustering of differential expression of *NAC* genes in different ripening stages of *S. lycopersicum*. The stage represents three different stages of *S. lycopersicum* fruit ripening; the source represents different sources of *NAC* genes.

The differential expression analysis of *NAC* genes at different maturation stages found that 7 *NAC* genes were differentially expressed (6 upregulated and 5 downregulated) in the MG vs. BR combination (Figure 7D). In the BR and BR10 combination (Figure 7E), this number was 5 (2 upregulated, 3 downregulated). In the MG vs. BR10 combination (Figure 7F), this number was 6 (3 up-regulated, 3 down-regulated). Although the number of differentially expressed *NAC* genes was low, their −Log10 (*p*-value) was relatively high (greater than 5) except for *Solyc05g055480*. To understand the expression patterns of *NAC* genes at different maturation stages, the expression levels of all *NAC* genes were clustered and displayed (Figure 8). For example, in the MG stage, *Solyc08g077110*, *Solyc06g063380*, *Solyc12g036480*, *Solyc04g025760*, *Solyc02g069960*, *Solyc07g066330*, *Solyc10g005010*, *Solyc09g010160* and *Solyc0 6g061080* are more active. In the Br stage, the expression of *Solyc11g068620*, *Solyc03g097650*, *Solyc03g1 15850* and *Solyc03g062750* was more active. In the BR10 stage, the expression of *Solyc03g083880*, *Solyc06g063430*, *Solyc07g062240*, *Solyc07g053680* and *Solyc10g079220* was more active. The expression results indicate that the *NAC* gene played a role in different maturation stages.

### 3.7. GO and KEGG Enrichment Analysis

**Figure 9:**
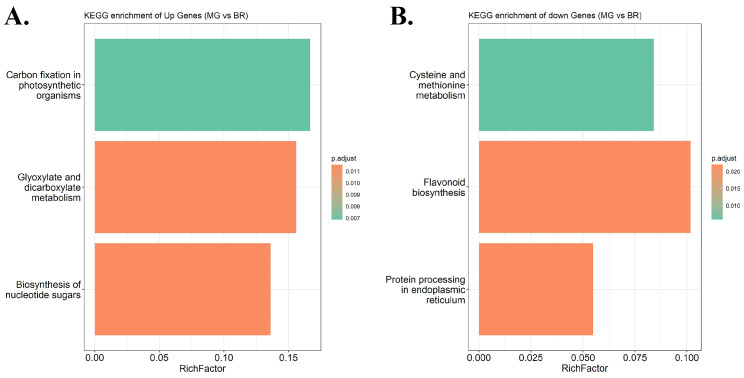
KEGG enrichment map of *S. lycopersicum* MG vs. BR. Horizontal coordinate RichFactor is the ratio of the number of genes enriched by *S. lycopersicum* in a pathway to the total number of genes in that pathway, and the vertical coordinate is the different pathways defined by KEGG. (**A**) Enrichment results of upregulated genes in KEGG. (**B**) Enrichment results of downregulated genes in KEGG.

Gene ontology (GO) can classify genes from three levels (MF: molecular function, BP: biological process, CC: cellular components), and predict the pathway of their function [56,57]. The differentially expressed genes of the three comparative combinations were extracted and subjected to GO enrichment analysis (Tables S7 and S8). Among the upregulated genes, genes in the combination of MG and BR were fully enriched in biological process and had lower fold enrichment, while the genes in the combinations of BR and BR10, and MG and BR10 were scattered into the three GO-enriched classes and had higher fold enrichment (Figure S7). Among the downregulated genes, genes of the BR and BR10 combination were enriched into two GO classes (BP and MF), while genes of the MG and BR, and MG and BR10 combinations were scattered into three GO classes (Figure S8). Taking the differentially expressed genes of the MG and BR10 combination as an example (Figures S7 and S8), the upregulated genes were highly enriched in several growth-essential pathways such as photorespiration, carbon fixation, glycogen metabolic process, and energy reserve metabolic process. The downregulated genes were highly enriched in structurally related pathways such as fruit ripening, oxidoreductase activity, and response to unfolded protein. The Kyoto Encyclopedia of Genes and Genomes (KEGG) can analyze the pathway enrichment of candidate genes in 7 categories to observe their interactions in different biochemical processes. Taking the MG and BR combination as an example, the upregulated genes were highly enriched in three pathways, the biosynthesis of nucleotide sugars, carbon fixation in photosynthetic organisms, and glyoxylate and dicarboxylate metabolism (Figure 9A, Table S9), which is similar to the results of GO. Downregulated genes were highly enriched in three additional pathways: protein processing in the endoplasmic reticulum, cysteine and methionine metabolism, and flavonoid biosynthesis (Figure 9B, Table S10). The results of differential expression show that upregulated genes were highly enriched in multiple active pathways such as maintaining organism survival and growth, while downregulated genes tended to be highly enriched in fruit ripening and protein structure maintenance.

The same GO and KEGG enrichment operations were performed on the *NAC* genes of *S. lycopersicum.* Due to the small number of *NAC* genes, only GO enrichment results were obtained (Figure S10, Table S11). The results show that 6 *NAC* genes were enriched in 7 BPs involved in growth and development, of which *Solyc05g007770.3.1* was present on all 7 BPs.

## 4. Discussion

In this study, 85 and 88 *NAC* genes were identified in *S. lycopersicum* and *S. pennellii*, respectively, which differed from the number identified in PlantTFDB v5.0 (101 and 105 *NAC* genes) [19]. There may be two reasons for this difference. First, in the process of identifying *NAC* genes, this study set two strict search thresholds, which resulted in fewer matches (see Section 2). Second, the tomato genome version used in PlantTFDB v5.0 is ITAG 2.3, and the latest ITAG 4.0 version was used in this study [31]. The difference in the number of *NAC* genes in *S. lycopersicum* may have been influenced by all two reasons, and this difference in *S. pennellii* may have mainly been due the first reason. Chromosomal mapping reveals that the NAC family appeared to be unevenly distributed across the two tomato genomes, and this uneven distribution is also seen in *Pyrus bretschneideri* (white pear) [58], *Betula pendula* (white birch) [59] and *Zea mays* L. (maize) [60] and other plant genomes.

On the basis of gene collinearity analysis, the origin of the NAC family since two paleohexaploidizations was analyzed. Our study reveals that most of the *NAC* genes in tomatoes originated from DWGT, and a small proportion of *NAC* genes originated from SWGT (Table 3). By linking paleohexaploidization to some basic features of the NAC family, we found many interesting results. The *NAC* genes of DWGT origin were unevenly distributed on all 12 chromosomes, while the *NAC* genes of SWGT origin were distributed centrally on chromosomes 2, 3, 5, 6, and 8 in the tomato (Figure 1 and Figure S1). This may have been because each paleohexaploidization was accompanied by genomic rearrangement, so the *NAC* genes of DWGT origin underwent two rearrangements and showed uneven distribution. Comparing the physicochemical properties of NAC proteins from different sources, the NAC proteins derived from SWGT were smaller than those produced by DWGT in terms of amino acid number, relative molecular mass, isoelectric point, and instability coefficient, and only the fat coefficient was slightly higher than the latter (Figure 2 and Table S1). This may have also been the result of two rearrangements, such as the distribution of *NAC* genes on chromosomes. This indicates that the two paleohexaploidization had different effects on the physicochemical properties of NAC proteins.

The phylogenetic trees of the NAC family in *S. lycopersicum* and *S. pennellii* share two striking similarities. One is that the evolutionary trees of both tomato NAC families can be divided into three subgroups, and the other is that each evolutionary tree had a subgroup with a high evolution rate. (Figure 4 and Figure S3). Combining the origin of *NAC* genes with their phylogenetic trees revealed that *NAC* genes of SWGT origin were most abundant in the subgroup with the fastest evolutionary rate, For example, 50% (5/10) of the *NAC* genes of SWGT origin were in the fastest evolving subgroup in *S. lycopersicum*. Similarly, nearly half (9/19) of the *NAC* genes in *S. pennellii* showed this phenomenon. Another phenomenon is that SWGT-derived *NAC* genes on other subgroups also tend to be close to the branches with faster evolutionary rates. This suggests that recent paleopolyploidization events can significantly increase the evolutionary rate of the NAC family.

**Figure 10:**
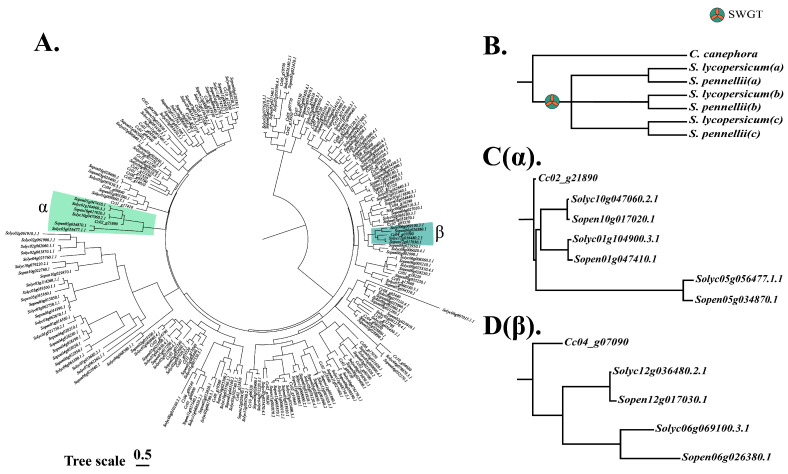
Phylogenetic tree and model of *C. canephora*, *S. pennellii*, and *S. lycopersicum NAC* genes. (**A**) Phylogenetic tree of *NAC* genes in the three species. (**B**) Phylogenetic model of the three species, *C. canephora*:*S. pennellii*:*S. lycopersicum* = 1:3:3. (**C**) Phylogenetic tree of the α branch in (**A**), consistent with model B. (**D**) The Phylogenetic tree of the β branch in A with gene loss in both tomatoes.

The analytical results of the genomic collinearity method and topological method also revealed interesting phenomena. After species divergence and paleohexaploidization, the offspring gene ratio of an ancestral gene should be *C. canephora*:*S. pennellii*:*S. lycopersicum* = 1:3:3 (Figure 10B), which was confirmed in the phylogenetic tree of all *NAC* genes in the three species (Figure 10C). However, gene clusters that fit the complete model are rare, and more gene clusters fit the residual model (Figure 10D). It is possible that the rearrangements following paleopolyploidization were also accompanied by substantial gene loss, resulting in a large reduction in family genes. This also explains why the number of *NAC* genes in *S. pennellii* and *S. lycopersicum* was less than three times the number of *NACs* in *C. canephora* (Table 1).

Several studies have shown that NAC transcription factors play a key role in the tomato fruit-ripening regulatory network. During fruit ripening, NAC transcription factors can act as positive regulators to promote the synthesis of hormones, such as ethylene and abscisic acid [12,15,16]. However, the expression of *NAC* genes can also accelerate leaf senescence and reduce photosynthesis time, thus reducing fruit quality [11]. The results of expression analysis in *S. lycopersicum* show that *NAC* genes are significantly up or down regulated at least once during fruit ripening (Figure 8). Most of the *NAC* genes showed significantly high expression at the MG and BR stages, i.e., promoting fruit ripening [16]. A small number of *NAC* genes showed significant expression at the BR10 stage, i.e., when tomatoes have ripened into soft fruits. The *NAC* gene remains highly expressed at the Br10 stage, probably to accelerate leaf senescence and terminate continued fruit growth [11]. This confirmed that the *NAC* gene can be expressed at different stages to promote and terminate fruit ripening in tomatoes [11,16]. Associating the origin of *NAC* genes with the results of enrichment analysis also revealed new phenomena. In the GO enrichment analysis of *NAC* genes in *S. lycopersicum*, a total of six genes were enriched in five pathways, five of which originated from DWGT and were more preferred to be enriched on processes related to plant development (e.g., leaf, system, phyllome, etc.) (Table S11), which was consistent with the results of the differential expression analysis. Expression analysis based on gene origin revealed that *NAC* genes of DWGT origin were more preferred to be expressed in the early and middle stages of fruit ripening, while *NAC* genes of SWGT origin were more preferred to be expressed in the early and late stages. That is, *NAC* genes of different origins perform functions at different stages. These results indicate that the DWGT-derived *NAC* gene was mainly expressed at the early stage of fruit ripening and promoted fruit ripening. However, SWGT-derived *NAC* genes were mainly expressed in late fruit ripening, terminating fruit ripening.

## 5. Conclusions

NAC transcription factors are among the important regulators of tomato fruit ripening. In this study, a novel analysis associating paleopolyploidization with the NAC family was performed in the tomato and its wild relatives. Genomewide collinearity analysis revealed that DWGT and SWGT were the main causes of NAC family amplification. We found that the two types of NAC family members derived from DWGT and SWGT exhibited different characteristics in terms of physicochemical properties, gene location, and phylogeny. We speculate that this difference is due to the fact that NAC family members derived from SWGT experienced more rearrangement events. For example, *NAC* genes derived from SWGT exhibit more concentration on chromosomes, smaller mean values in physicochemical properties, and faster evolutionary rates. Analysis of paleohexaploidization and gene expression showed that *NAC* genes derived from DWGT tend to be expressed at early and mid-fruit ripening and promote fruit ripening. In contrast, SWGT-derived *NAC* genes tended to be expressed at late fruit ripening, promoting leaf senescence and terminating fruit growth. Our study links paleohexaploidization to the NAC family and provides a scheme to explore the link between the origin of the NAC family and its function. This study also provides guidance for the developmental control of tomato fruits. We hope to continue to apply our research ideas and methods to more plant genes. Meanwhile, the linkage between paleopolyploidization and gene families needs more studies to be confirmed.

## Figures and Tables

**Table 1:** Identification information of *NAC*.

Species Name	Data Version	Chromosome Number	Total Gene Number	NAC Candidate Number
*Coffea canephora*	AUK _v1	2n = 22	25,574	60
*Solanum lycopersicum*	ITAG 4.0	2n = 24	34,075	85
*Solanum pennellii*	Spenn-v2.0	2n = 24	48,923	88

**Table 2:** Statistical table of the number of genes from different sources.

Species Name	Count Type	Singleton	Dispersed	Proximal	Tandem	SWGT	DWGT
*C. canephora*	Number	7153	8354	300	659	——	9108
	Percentage	27.97%	32.67%	1.17%	2.58%	——	35.61%
*S. lycopersicum*	Number	5784	10,794	1672	4012	3254	14,394
	Percentage	16.97%	31.68%	4.91%	11.77%	9.55%	42.24%
*S. pennellii*	Number	3184	7054	1035	7556	20,861	14,027
	Percentage	6.51%	14.42%	2.12%	15.44%	42.64%	28.67%

Segmental: match genes in syntenic blocks; tandem: continuous repeat; proximal: in nearby chromosomal region but not adjacent; dispersed: other modes than segmental, tandem, and proximal. Number: number of genes by type; percentage (%): percentage between the number of each gene type and the total number of genes in the whole genome.

**Table 3:** Information on the source of the *NAC* gene.

Species Name	Count Type	Tandem	SWGT	DWGT
*C. canephora*	Number	1	——	38
	Percentage	1.67%	——	63.33%
*S. lycopersicum*	Number	3	10	61
	Percentage	3.53%	11.76%	71.76%
*S. pennellii*	Number	7	19	57
	Percentage	7.95%	21.59%	64.77%

Number: Number of NAC genes produced by different sources; percentage (%): percentage of the number of NAC genes produced by each source to the total number of NAC genes of the corresponding species.

**Table 4:** Information on the source of the *NAC* gene.

Species Name	Count Type	SWGT	DWGT	*NAC* SWGT	*NAC* DWGT
*C. canephora*	Ka/Ks < 1	——	2692	——	13
	Ka/Ks > 1	——	545	——	1
*S. lycopersicum*	Ka/Ks < 1	339	778	1	4
	Ka/Ks > 1	363	1546	0	10
*S. pennellii*	Ka/Ks < 1	6947	3065	4	19
	Ka/Ks > 1	823	245	2	2

Count type represents different selection pressure directions (columns) and different source gene pair types (rows); numbers represent the number of collinear gene pairs.

## Data Availability

Data are contained within the article or available upon reasonable request from the corresponding author.

## Supplementary Materials

The following supporting information can be downloaded at: https://www.mdpi.com/article/10.3390/life12081236/s1, Figure S1: physical and chemical properties of two tomato NAC proteins. Figure S2: statistical chart of physicochemical properties of *Solanum pennellii* NAC protein. Figure S3: *Solanum pennellii* NAC protein phylogenetic tree. Figure S4: *Coffea canephora* NAC protein phylogenetic tree. Figure S5: collinear map of *Solanum pennellii* NAC gene. Figure S6: collinear map of *Coffea canephora NAC* gene. Figure S7: GO enrichment dot plot of Solanum lycopersicum upregulated genes in different periods. Figure S8: GO enrichment map of *Solanum lycopersicum* downregulated genes in different periods. Figure S9: KEGG enrichment map of *Solanum lycopersicum* upregulated genes in different periods. Figure S10: GO enrichment map of *NAC* genes in *Solanum lycopersicum* in different periods. Table S1: physicochemical properties of two tomato NAC proteins. Table S2: secondary structure prediction table of two tomato NAC proteins. Table S3: selection pressure gauge among different WGT-producing genes. Table S4: selection pressure gauge among *NAC* genes produced by different WGT. Table S5: differential expression results of *Solanum lycopersicum* gene in different periods. Table S6: differential expression results of *Solanum lycopersicum NAC* gene in different periods. Table S7: GO enrichment results of downregulated genes in *Solanum lycopersicum* in different periods. Table S8: GO enrichment results of upregulated genes in *Solanum lycopersicum* in different periods. Table S9: KEGG enrichment results of downregulated genes in *Solanum lycopersicum* in different periods. Table S10: KEGG enrichment results of upregulated genes in *Solanum lycopersicum* in different periods. Table S11: GO enrichment results of *Solanum lycopersicum NAC* gene in different periods.

## Abbreviations

The following abbreviations are used in this manuscript:

TFs	Transcription factors
WGD	Whole-genome duplication
WGT	Whole-genome triplication
mya	Million years ago
DWGT	Whole-genome triplication event shared by dicots
SWGT	Whole-genome triplication event shared by Solanaceae
MG	Mature green
Br	Breaker
Br10	Breaker + 10 days
