# Supplementary figures and images for: Genome-Wide Analysis of the NAC Family Associated with Two Paleohexaploidization Events in the Tomato
Source: Life (Basel). 2022 Aug 15;12(8):1236. doi: 10.3390/life12081236 (PMC9410287; doi:10.3390/life12081236)

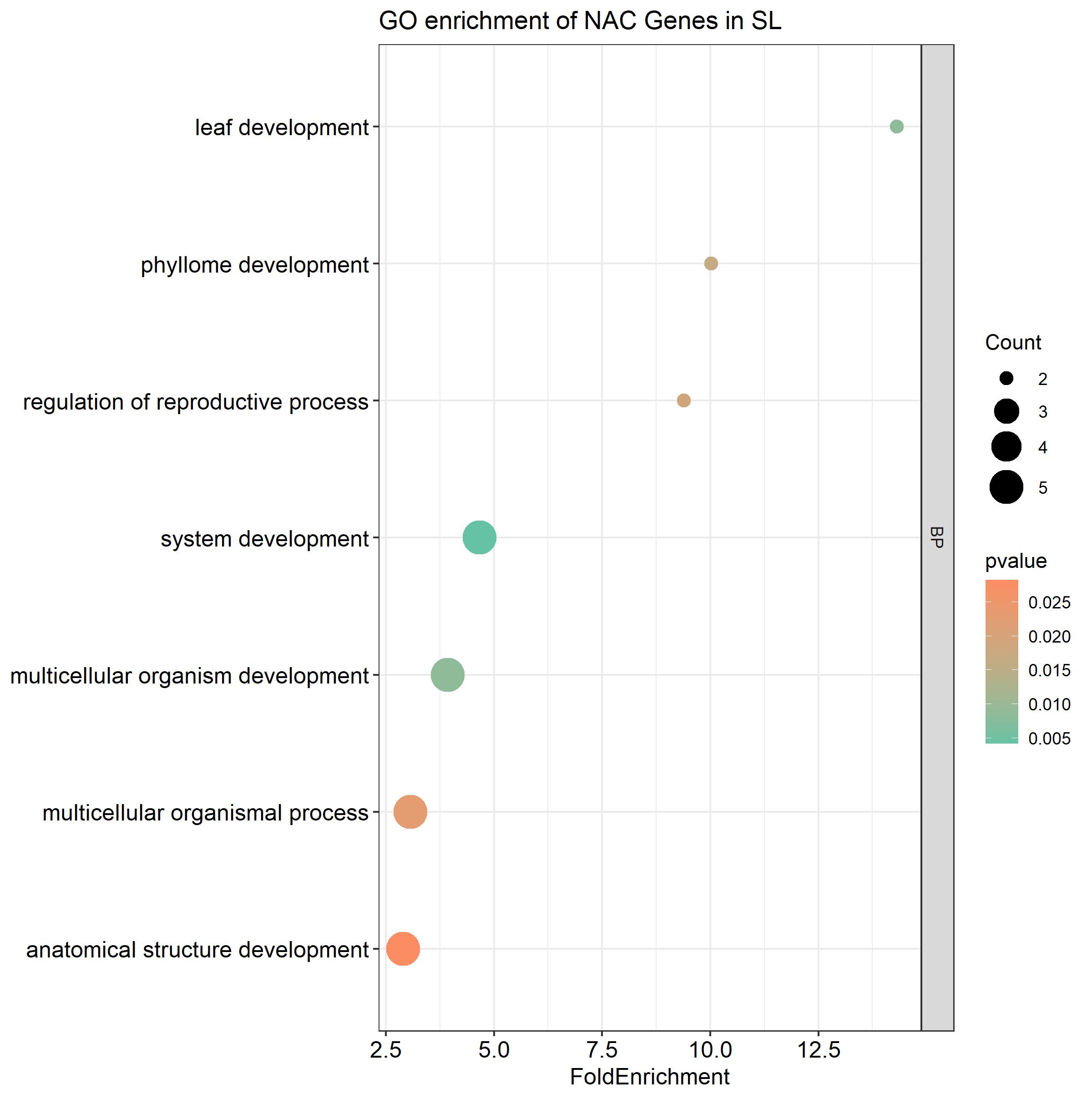

Supplement: Supplementary file 1 [file life-12-01236-s001.zip › life-1834936-supplementary/Supplementary Figure/Figure S10(GO enrichment map of NAC genes in Solanum lycopersicum in different periods).jpg]

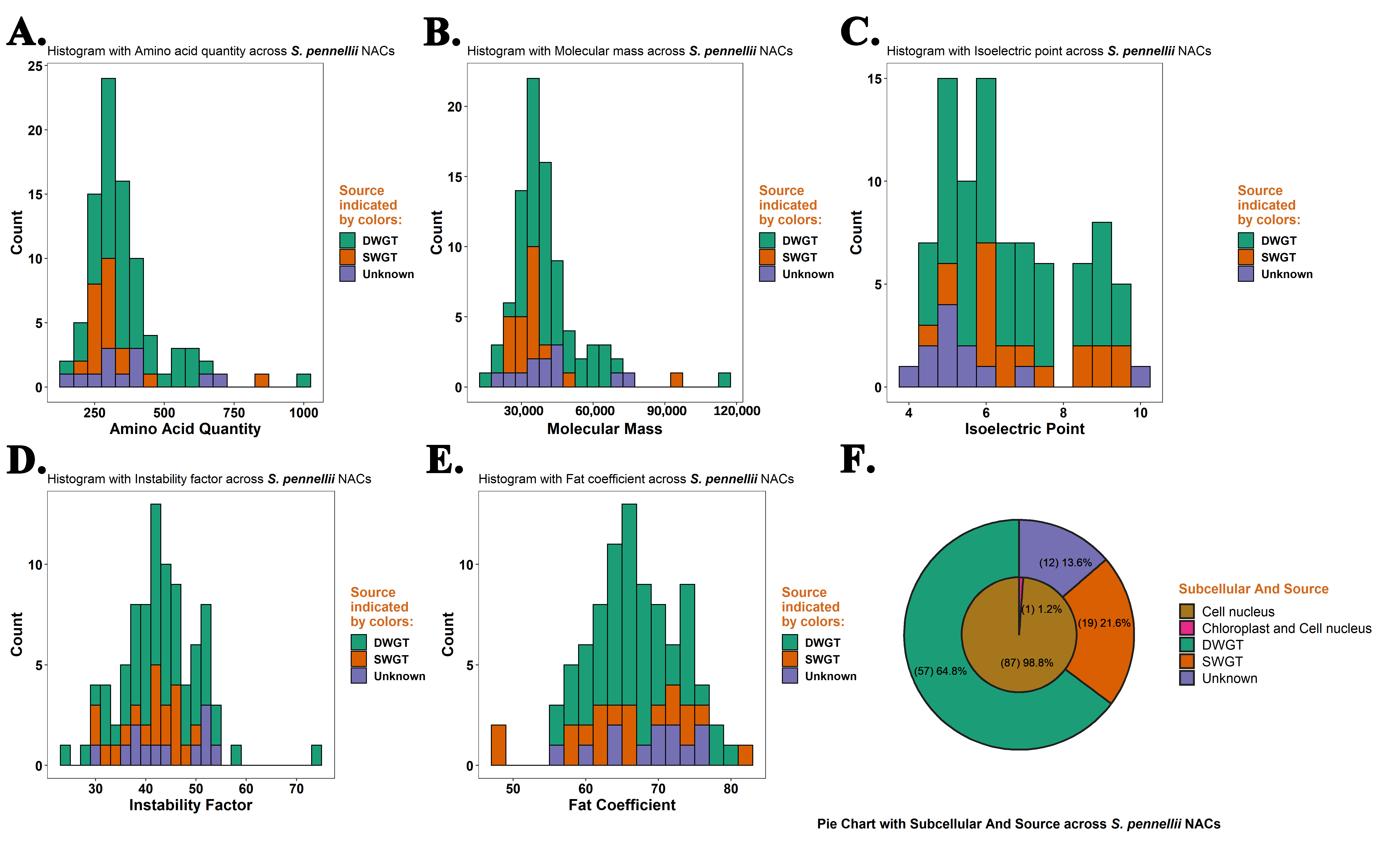

Supplement: Supplementary file 1 [file life-12-01236-s001.zip › life-1834936-supplementary/Supplementary Figure/Figure S2(Statistical chart of physicochemical properties of Solanum pennellii NAC protein.).png]

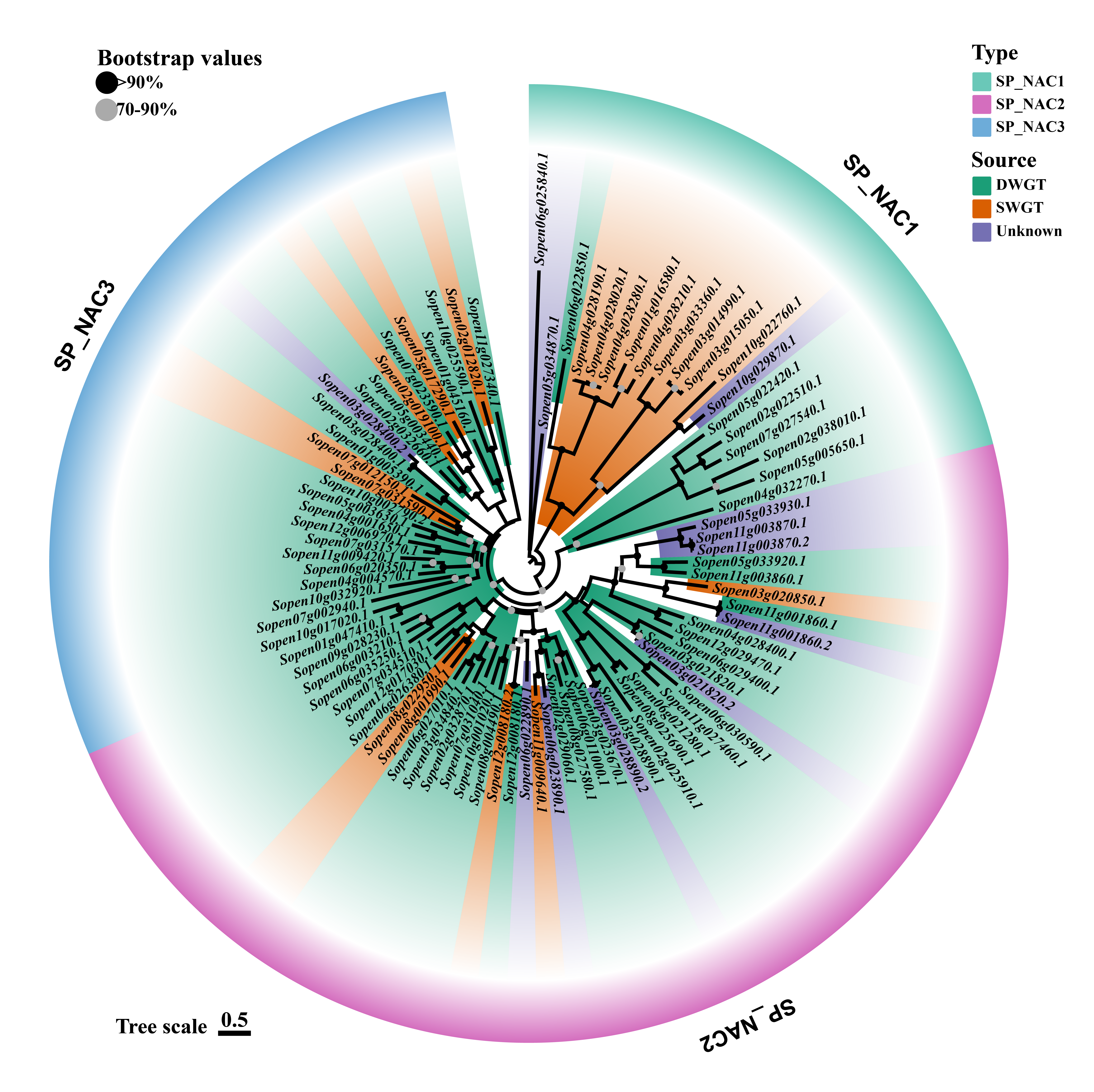

Supplement: Supplementary file 1 [file life-12-01236-s001.zip › life-1834936-supplementary/Supplementary Figure/Figure S3(Solanum pennellii NAC protein phylogenetic tree).png]

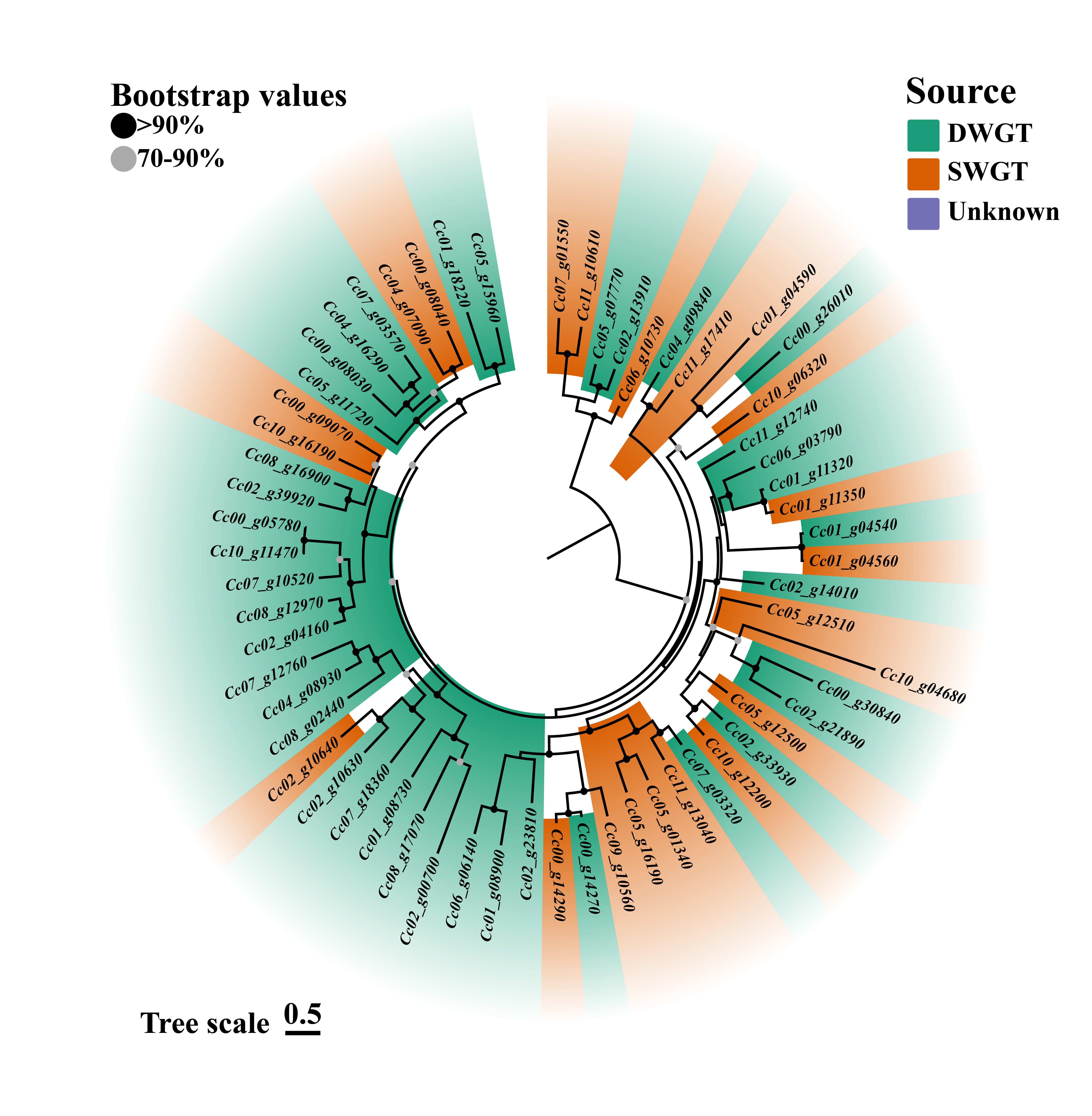

Supplement: Supplementary file 1 [file life-12-01236-s001.zip › life-1834936-supplementary/Supplementary Figure/Figure S4(Coffea canephora NAC protein phylogenetic tree).png]

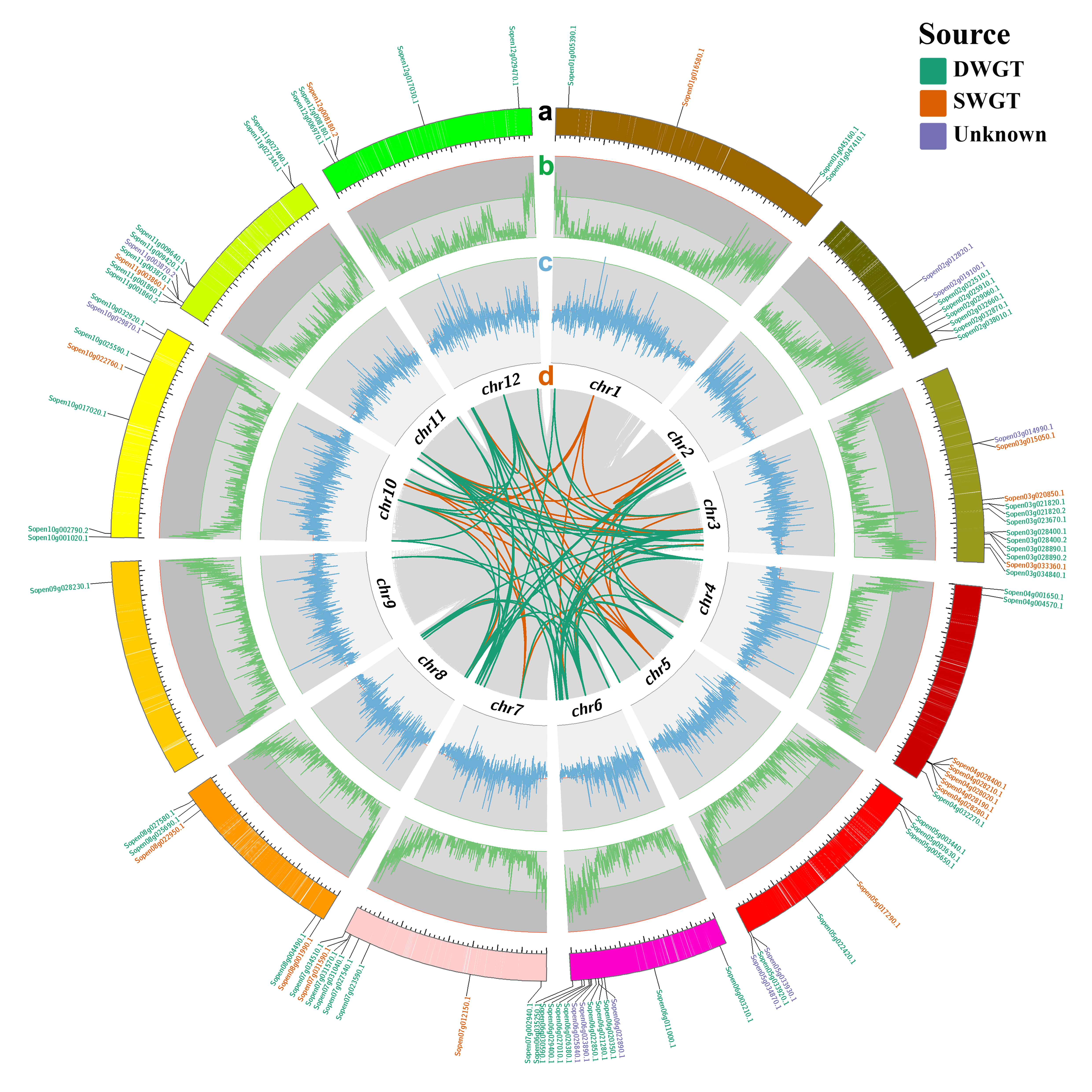

Supplement: Supplementary file 1 [file life-12-01236-s001.zip › life-1834936-supplementary/Supplementary Figure/Figure S5(Collinear map of Solanum pennellii NAC gene).png]

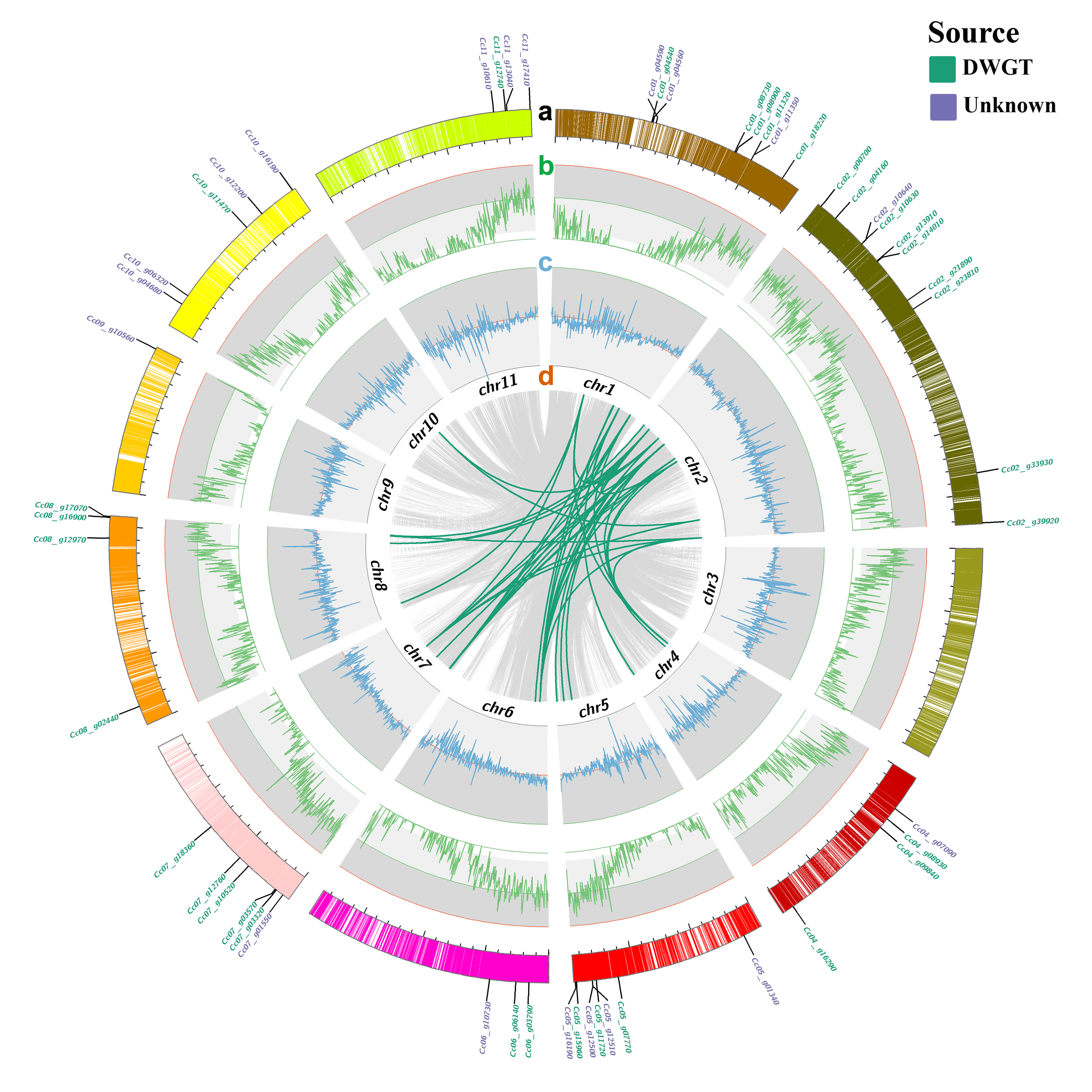

Supplement: Supplementary file 1 [file life-12-01236-s001.zip › life-1834936-supplementary/Supplementary Figure/Figure S6(Collinear map of Coffea canephora NAC gene).png]

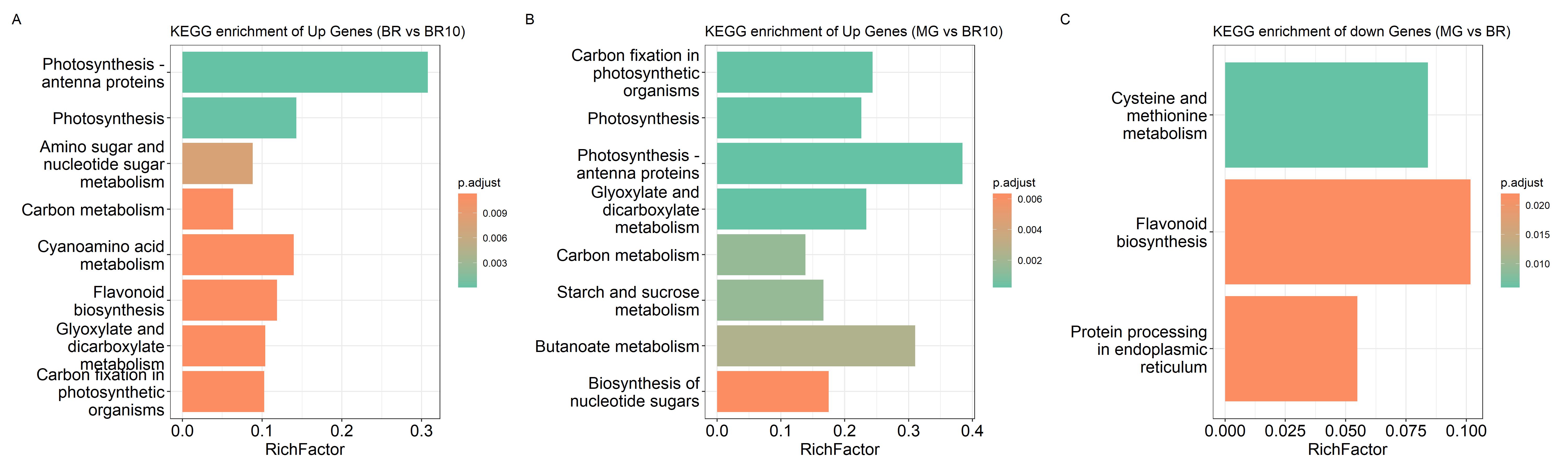

Supplement: Supplementary file 1 [file life-12-01236-s001.zip › life-1834936-supplementary/Supplementary Figure/Figure S9(KEGG enrichment map of Solanum lycopersicum up-regulated genes in different periods).jpg]
